# p16^INK4a^/Ki-67 dual stain cytology for cervical cancer screening in Thika district, Kenya

**DOI:** 10.1186/s13027-015-0020-2

**Published:** 2015-08-11

**Authors:** Caroline Wangari Ngugi, Dietmar Schmidt, Karanja Wanyoro, Hamadi Boga, Peter Wanzala, Anne Muigai, John Mbithi, Magnus von Knebel Doeberitz, Miriam Reuschenbach

**Affiliations:** College of Health Sciences, Jomo Kenyatta University of Agriculture and Technology, Nairobi, Kenya; Institute of Pathology, A2.2 Mannheim, Germany; Thika District Hospital, Thika, Kenya; Faculty of Science, Jomo Kenyatta University of Agriculture and Technology, Nairobi, Kenya; Centre for Public Health Research- Kenya Institute of Medical Research, Nairobi, Kenya; Department of Medical Laboratory Sciences, Kenyatta University, Nairobi, Kenya; Department of Applied Tumor Biology, Institute of Pathology, University of Heidelberg, and Clinical Cooperation Unit, German Cancer Research Cancer (DKFZ), 69120 Heidelberg, Germany

**Keywords:** Cervical cancer, Developing countries, Screening, HPV, p16^INK4a^, Ki-67

## Abstract

**Background:**

The identification of suited early detection tests is one among the multiple requirements to reduce cervical cancer incidence in developing countries.

**Methods:**

We evaluated p16^INK4a^/Ki-67 dual-stain cytology in a screening population in Thika district, Kenya and compared it to high-risk human papillomavirus (HR-HPV) DNA testing and visual inspection by acetic acid (VIA) and Lugol’s iodine (VILI).

**Results:**

Valid results for all tests could be obtained in 477 women. 20.9 % (100/477) were tested positive for HR-HPV DNA, 3.1 % (15/477) had positive VIA/VILI and 8.2 % (39/477) positive p16^INK4a^/Ki-67 cytology. Of 22 women that showed up for colposcopy and biopsy, 6 women were diagnosed with CIN3 and two with CIN2. All women with CIN2/3 were negative in VIA/VILI screening and positive by HR-HPV DNA testing. But HPV was also positive in 91.7 % (11/12) of women with normal histology. p16^INK4a^/Ki-67 cytology was positive in all 6 women with CIN3, in one of the two CIN2 and in only 8.3 % (1/12) of women with normal histology.

**Conclusions:**

p16^INK4a^/Ki-67 cytology is an interesting test for further studies in developing countries, since our findings point to a lower fraction of false positive test results using p16^INK4a^/Ki-67 cytology compared to HPV DNA testing in a Kenyan screening population. VIA/VILI missed all histology-proven CIN2/3.

## Background

Cancer of the uterine cervix is the third most common cancer in women around the world and still the leading cancer in women in Sub Sahara Africa [1]. Of the 450,000 new cases every year, over 80 % occur in resource poor countries [1]. In Kenya, the annual crude incidence rate of cervical cancer is 16.5 per 100,000 women with a leading mortality rate of 13.5 in 100,000 women [1, 2].

Implementation of nationwide screening for cervical cancer has been successful in reducing the incidence and mortality rates of cervical cancer in many countries [3]. A further reduction in disease incidence is suggested to be reached by prophylactic HPV vaccinations, if they are combined with continued screening [4]. However, screening and vaccination coverage in developing countries is dramatically low. In Kenya, only 3.2 % of the female population aged 18–69 have been screened for cervical cancer [2].

Cervical cancer is caused by long lasting infections of the cervical squamous cell epithelium with human papillomaviruses (HPV). The infection is usually acquired during adolescence when women become sexually active and infection prevalence my reach over 50 % [5]. While the vast majority of women clear the infection, upon persistent HPV infections premalignant alterations (cervical intraepithelial neoplasia, CIN) may occur after several years, which precede the development of cervical cancer. Cervical cancer is largely preventable by detecting premalignant alterations and treat them by ablation or excisional means [5].

Currently the primary screening tests for cervical cancer in most countries with implemented screening programs is Pap cytology, where a smear is taken from the cervix, stained with a dye cocktail and assessed under a light microscope for neoplastic alterations. Pap screening requires advanced infrastructures and experience as it is characterized by multiple visits for diagnosis and treatment, because it has a low single-test sensitivity of between 55 to 80 % [6, 7] and generates many equivocal results which need further work-up [8]. Before women are referred to treatment, classically by removal of the affected tissue (conization), the test is repeated, the cervix is assessed visually by colposcopy and diagnostic biopsies are taken. Overall Pap cytology-based screening has frequently proven challenging and unworkable in low-resource settings [9].

Different programs investigate and assess alternative approaches, particularly the use of visual screening methods, such as visual inspection with acetic acid (VIA), for pre-cancer and cancer detection and the use of cryotherapy as a pre-cancer treatment method. This is optimally achieved in a single visit and can be carried out also by competent non-physicians, including nurses and midwives. Because the diagnostic accuracy of VIA is largely heterogeneous, in some setting only reaching a sensitivity of ~40 % [10] and thereby missing women with pre-cancer that would need treatment, still alternative tests suited for rural and limited resource conditions are currently designed and should be scientifically evaluated.

Detecting the viral DNA is a highly sensitive test for cervical cancer early detection, detecting more premalignant alterations than Pap cytology, used in addition to Pap cytology in certain settings and currently introduced as an alternative for primary screening in many industrialized countries [11]. However, considering the high prevalence of HPV infections, particularly in young women, detecting HPV DNA is a poorly specific test for real cellular alterations and therefore requires additional triage tests for the specific identification of women needing further work-up or treatment [12]. Much effort is currently spent in evaluating the clinical performance of new biomarkers, in order to reduce referral rates, unnecessary treatments and finally costs. In developing countries new biomarkers are particularly attractive as potential point-of-care tests. They can be used to specifically identify directly at one visit those women that need treatment and may thereby reduce the lost-to-follow-up rate [13].

There are different biomarkers that have been identified and suggested to improve diagnosis of cervical cancer and its precancerous lesions. One promising marker is the cyclin dependent kinase inhibitor p16^INK4a^, which becomes overexpressed in response to viral oncogene E7 expression [14]. A number of studies have investigated the clinical use of p16^INK4a^ as a specific biomarker for cells with transforming HPV infections [15]. HPV-transformed cells overexpress p16^INK4a^ but retain the capacity to proliferate and are thus double-positive for p16^INK4a^ and the proliferation marker Ki-67, while senescent cells express p16^INK4a^ but are Ki-67 negative [16]. An immunochemical test which in parallel detects p16^INK4a^ and the proliferation marker Ki-67 (p16^INK4a^/Ki-67 cytology, CINtec PLUS) is designed for application on cytologic smears taken from the uterine cervix with a brush, spread on a glass slide, stained for expression of the two markers and assessed under a light microscope. The test demonstrated high sensitivity comparable to HPV DNA testing but superior specificity compared to HPV DNA testing in a European screening population [15, 17].

The objective of the present study was to study overall positivity rates of p16^INK4a^/Ki-67 cytology in a screening population of Kenyan women and compare it to VIA/VILI and HPV DNA testing. Additionally the study was designed to evaluate the diagnostic performance of the tests to detect high grade CIN.

## Results

### Characteristics of the study population and proportion of positive VIA/VILI, HPV and p16^INK4a^/Ki-67 results

A total of 498 women met the eligibility criteria. After exclusion of women with incomplete personal information (no age documented), no VIA/VILI and/or invalid laboratory results in either Pap cytology (low cell number), p16^INK4a^/Ki-67 cytology (low cell number) and/or HPV DNA (no cell pellet), 477 samples remained for evaluation. Mean age of these 477 study participants was 35.6 years, ranging between 18 and 74 years (standard deviation 9.9 years). A detailed socio-demographic characterization of the study population is given in reference [18].

**Fig. 1:**
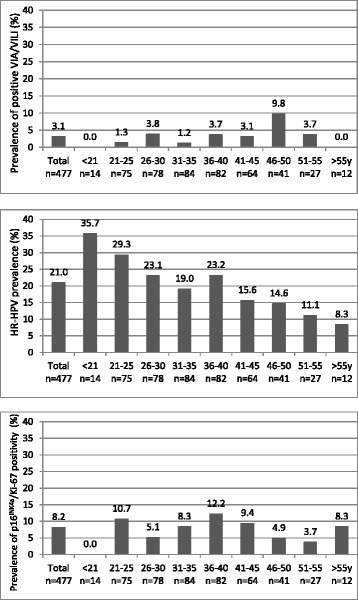
Proportion of positive VIA/VILI, HR-HPV tests and positive p16^INK4a^/Ki-67 dual-stain cytology in different age groups

p16^INK4a^/Ki-67 cytology was positive in 8.2 % (39/477) of the women, with highest rate of positivity in women of age 36 to 40 years (12.2 %, 10/82) and no positives in women younger than 21 years (Fig. 1).

Of the 477 women, 20.9 % (100/477) were tested positive for HR-HPV. Highest HPV prevalence was found with 35.7 % (5/14) among women younger than 21 years (18–20 years) and prevalence decreased with older age groups with the lowest prevalence (8.3 %, 1/12) in those older than 55 years (Fig. 1).

VIA/VILI was only positive in a total of 3.1 % (15/477) of the women with the highest prevalence in the age group 46 to 50 years (9.8 %, 4/41) and no positives in women younger than 21 or older than 55 years (Fig. 1).

Most women had normal cytology (89.3 %, 426/477), 2.9 % (14/477) had LSIL, 0.2 % (1/477) AGUS, 5.5 % (26/477) ASCUS and 2.1 % (10/477) had HSIL diagnosed in Pap cytology (Table 1).

**Table 1:** Proportion of positive VIA/VILI, HPV DNA and p16^INK4a^/Ki-67 results in relation to Pap cytology and colposcopy/biopsy. Women with normal colposcopy that were not biopsied (n = 4) are included in the normal histology group

	Total n	VIA/VILI positive	HPV DNA positive	p16^INK4a^/Ki-67 positive
Colposcopy/histology				
Normal	12	0 (0.0 %)	11 (91.7 %)	1 (8.3 %)
CIN1	2	0 (0.0 %)	2 (100.0 %)	0 (0.0 %)
CIN2	2	0 (0.0 %)	2 (100.0 %)	1 (50.0 %)
CIN3	6	0 (0.0 %)	6 (100.0 %)	6 (100.0 %)
Not available	455	15	79	31
Pap cytology				
Normal	426	9 (2.1 %)	76 (17.8 %)	18 (4.2 %)
LSIL	14	0 (0.0 %)	11 (78.6 %)	7 (50.0 %)
AGUS	1	0 (0.0 %)	1 (100.0)	1 (100.0)
ASCUS	26	3 (11.5 %)	4 (15.4 %)	6 (23.1 %)
HSIL	10	3 (30.0 %)	8 (80.0 %)	7 (70.0 %)

### Correlation of VIA/VILI, HPV results and p16^INK4a^/Ki-67 cytology

Agreements between VIA/VILI, HPV results and p16INK4a/Ki-67 cytology are shown in Table 2. Poor agreement was found between VIA/VILI results and HPV or p16^INK4a^/Ki-67, respectively, with slightly better agreement between VIA/VILI and p16^INK4a^/Ki-67 than between VIA/VILI and HPV DNA testing (kappa −0.003 (HPV) and 0.030 (p16^INK4a^/Ki-67), respectively; overall positive and negative agreement 77.1 % (HPV) and 89.5 % (p16^INK4a^/Ki-67)). Of the 15 VIA/VILI positive women, only 3 were tested positive for HPV DNA and 2 positive for p16^INK4a^/Ki-67.

**Table 2:** Agreement between VIA/VILI, HPV results and p16^INK4a^/Ki-67 cytology. Shown are absolute numbers of positive and negative samples for the three tests

		p16^INK4a^/Ki-67		VIA/VILI	
		Negative	Positive	Negative	Positive
HPV DNA	Negative	373	4	365	12
	Positive	65	35	97	3
VIA/VILI	Negative	425	37		
	Positive	13	2		

A moderate agreement was observed between HPV and p16^INK4a/^Ki-67 results (kappa = 0.437, p < 0.001) with total positive and negative agreement of 85.5 %. The majority of samples positive for p16^INK4a/^Ki-67 were also positive for HPV DNA (35/39), however more than half of HPV DNA positive samples were p16^INK4a/^Ki-67 negative (65/100).

### Performance of the tests to detect high grade cervical intraepithelial neoplasia

The histopathology of a biopsy is considered as the gold-standard for the diagnosis of cervical dysplasia. Women were invited to colposcopy and biopsy if at least one of the tests VIA/VILI, Pap cytology, HPV DNA, and/or p16^INK4a^/Ki-67 was positive. Women with only negative test results were considered as disease negative. Of the 22 women which appeared for colposcopy, 4 had no visible lesion and were thus not biopsied, 8 had normal histology in the punch biopsies, 2 women had CIN1, 2 had CIN2 and 6 women were diagnosed with CIN3. All biopsies were evaluated independently from H&E evaluation for p16^INK4a^ expression and all CIN2 and CIN3 lesions showed positive diffuse p16^INK4a^ expression, while normal and CIN1 cases were p16^INK4a^-negative, thus supporting the diagnosis of high-grade dysplasia in 8 women (2 CIN2, 6 CIN3).

The 6 lesions diagnosed as CIN3 and the two lesions diagnosed as CIN2 in a punch biopsy were all not positive in VIA/VILI and thus would have been missed. HPV DNA testing detected all of these high grade lesions, but was also positive in 91.7 % (11/12) of women with normal histology. p16^INK4a^/Ki-67 cytology was positive in all 6 CIN3 lesions, in one of the two CIN2 lesions and in only 8.3 % (1/12) of women with normal histology in the punch biopsy (Table 1).

As an additional estimate of dysplasia detection rates, the proportion of positive VIA/VILI, HPV DNA and p16^INK4a^/Ki-67 results in relation to Pap cytology were assessed. VIA/VILI was positive in only 30 % (3/10) of women with HSIL cytology. HPV DNA testing was positive in 80 % (8/10) of women with HSIL cytology, but also positive in 17.8 % (76/426) of women with negative Pap cytology. p16^INK4a^/Ki-67 dual stain cytology was positive in 70 % (7/10) women with HSIL cytology and only 4.2 % (18/426) women with normal cytology (Table 1).

## Discussion

The high incidence of cervical cancer in Kenya and other developing countries is amongst other reasons due to lacking opportunities for early detection followed by treatment of pre-invasive lesions when necessary.

Visual inspection (VIA and VILI) is used in some setting with variable sensitivity and specificity [10]. HPV DNA testing is being evaluated in developing countries as a more objective test with higher sensitivity and limited specificity [19]. The aim of the present study was to evaluate p16^INK4a^/Ki-67 dual-stain cytology in a screening population of Kenyan women in comparison to HPV DNA testing and VIA/VILI. p16^INK4a^ is a marker with a potentially favorable diagnostic profile, i.e. low rate of false-positive test results and high detection rate of disease [17, 20].

HPV DNA testing yielded the highest rate of overall positive results (20.9 %; 100/477) in our study with highest HPV prevalence in women younger than 21 years. Among women with available colposcopy and biopsy HPV DNA testing detected all women with histologically confirmed high grade lesions (n = 8), but was also positive in 91.7 % (11/12) of women with normal histology. p16^INK4a^/Ki-67 cytology yielded a lower overall rate of positive results (8.2 %; 39/477) and most importantly no positives in women younger than 21 years. This indicates that the test does not pick up the many transient and non-transforming HPV infections in this age group that most likely do not need to be treated. Furthermore p16^INK4a^/Ki-67 cytology was positive in all 6 women with CIN3 lesions, in one of the two CIN2 lesions and in only 8.3 % (1/12) of women with normal histology in the punch biopsy.

In our study p16^INK4a^/Ki-67 cytology staining was performed in an experienced laboratory in Heidelberg, Germany. However, the availability as ready-to-use reagents and little technical equipment which is required might give the opportunity to implement it in an environment with less advanced infrastructure. We used the PreservCyt ThinPrep system for cytology preparations in the study as this allowed to use one cervical swab for all tests. However, p16^INK4a^/Ki-67 cytology staining can also be performed on conventional cytology smears. There is no published data on direct comparison of p16^INK4a^/Ki-67 cytology using both cytologic methods (liquid-based vs conventional) for the same woman; there was however no significant difference in p16^INK4a^/Ki-67 cytology performance between women screened with conventional smears compared to women screened with liquid-based cytology demonstrated in a large European primary screening study [17].

In the present study we solely evaluated the diagnostic performance of the tests; we however would like to point out that there is an urgent need now to complement diagnostic studies by well-designed cost-effectiveness analyses. Presently p16^INK4a^/Ki67, particularly in combination with the ThinPrep system cannot be regarded as affordable for low-resource settings. On the long run however cost-effectiveness will largely depend on incentives of ministries, industry and finally the performance of the used tests. In this regard the present study may add an important data set for upcoming initiatives and cost analyses.

VIA/VILI, which is currently used in some regions in developing countries, detected none of the women with histologically proven CIN2+, thus women would have been missed by the test. VIA/VILI has the advantage that it is cheap, requires no advanced equipment and can be used as a point-of-care test allowing for screening and cryotherapy if necessary on the same day. However its diagnostic performance can be poor, as indicated by our result.

## Conclusions

Although the sample size is limited by the low number of women which followed the invitation to colposcopy and biopsy, it is a promising finding that p16^INK4a^/Ki-67 testing yielded a similar detection rate of CIN2+ and a substantially lower rate of positive tests in women with negative histology compared to HPV DNA. Moreover, the overall lower test positivity rate particularly in young women of p16^INK4a^/Ki-67 compared to HPV DNA testing suggests that only little transient infections are picked up and warrants further evaluation of the test in countries with high HPV prevalence. p16^INK4a^/Ki-67 cytology can be performed within 5 hours manually and does not necessarily require technical equipment except a heating bath and a microscope, thus potentially being performed at a health facility within one day under standardized and controlled conditions. Assessing the slides does not require morphological interpretation, positive are considered all slides with one or more cells expressing both markers, p16^INK4a^ and Ki-67. This can be learned in a brief training session even by people without prior cytologic or pathologic training [21]. For further studies also automated software-based evaluations of the slides are well conceivable [22].

## Methods

### Study design

The rate of positive test results and diagnostic performance of VIA/VILI, HR-HPV DNA detection and p16^INK4a^/Ki-67 dual-stain cytology was evaluated in a cross-sectional study in women living in Thika district, Kenya. Liquid-based cytology (LBC) samples were taken and sent to Heidelberg, Germany, where HR-HPV DNA testing and p16^INK4a^/Ki-67 immunostaining was performed. Additionally, Pap staining was performed from the LBC sample in Heidelberg. According to the study protocol, women with a positive VIA/VILI and/or HR-HPV and/or p16^INK4a^/Ki-67 test, and/or Pap cytology result were invited for colposcopy/biopsy to obtain a histopathologic diagnosis followed by treatment in cases were indicated. With the limitation that a sole positive HR-HPV DNA test only resulted in immediate colposcopy in women older than 30 years.

Ethical approval was obtained from the National Ethical Review Committee at the Kenya Medical Research Institute and women who agreed to participate signed an informed consent form.

### Recruitment of women and cervical sampling

Women were recruited for the study in Thika District, Kenya in February 2010. The population is both rural and urban. Women who live in this region were invited to participate in the study through posters placed at markets places, churches, and health centers. Women were eligible for the study if they were (a) self-identified as residing in the region, (b) were ages 18 and above, (c) had an intact uterus, (d) did not report the use of vaginal medication for the previous two days, (e) did not report treatment for cervical disease for the previous 6 months, and (f) were not pregnant at the time of the study.

Cervical cells were collected by female nurses using the liquid-based cytology ThinPrep® Pap test Kit (Hologic, Bedford, USA) according to manufacturer’s instruction and stored in the PreservCyt solution vial (Hologic, Bedford, USA) at ambient temperature. All tests, Pap cytology, p16^INK4a^/Ki-67 cytology, and HR-HPV DNA detection were performed from the same vial at the Department of Applied Tumor Biology, Heidelberg, Germany. VIA/VILI was performed by the nurses after the cytology sample was taken and documented in a standard form.

### Pap cytology

ThinPrep® slides were stained according to routine Pap staining laboratory protocols. All Pap cytology slides were read blinded to all other results at the Institute of Pathology, A2, Mannheim, Germany by an experienced cytotechnologist and gyneco-pathologist. The result was reported according to the Bethesda system.

### p16^INK4a^/Ki-67 dual-stain cytology

A second ThinPrep® slide was prepared from all samples and stained manually with a mix of two monoclonal antibodies against p16^INK4a^ and Ki-67, respectively using the CINtec PLUS kit (Roche mtm Laboratories, Heidelberg, Germany) according to the manufacturer’s instructions. The cervical cancer cell line C4-1 was used as positive control. The dual-staining results in brown visualization of p16^INK4a^ protein and red visualization of Ki-67 protein. Samples were considered as p16^INK4a^/Ki-67 dual-stain positive when at least one cell was visible on the slide under a light microscope expressing both proteins, thus if at least one cell was found with a red nucleus (Ki-67) surrounded by brown cytoplasm (p16^INK4a^).

### High-risk human papillomavirus detection

After processing for cytology, four mls of residual PreservCyt® solution was used for HR-HPV detection. The HR-HPV detection was carried out using the Hybrid capture 2® (hc2) test (Qiagen, Hilden, Germany) according to the manufacturer’s instructions. A positive hc2 result was defined as RLU/Co ≥ 1.

### Colposcopy, biopsy and histology

Colposcopy was performed at Thika District Hospital by an experienced gynecologist. Four to five punch biopsies were taken from the cervix of women with visible lesions. The tissue was placed in formalin and embedded in paraffin for hematoxylin & eosin staining. The slides were assessed by an experienced pathologist at Institute of Pathology, A2, Mannheim, Germany. A second section from all biopsies was stained immunohistochemically for p16^INK4a^ (CINtec histology, mtm Laboratories, Heidelberg) and slides with a diffuse p16^INK4a^ expression beginning in the basal and parabasal cell layers were considered as p16^INK4a^ histology positive.

### Data evaluation and disease endpoint

Results were computed as number of positive and negative test results in the entire study cohort, in different age groups and separated for cyologic and histologic diagnosis. Inter-assay agreements were assessed using Cohen's kappa statistics.

Women with negative VIA/VILI, HPV DNA, p16^INK4a^/Ki-67 and Pap cytology test were considered as disease negative. Women with a positive VIA/VILI and/or HR-HPV and/or p16^INK4a^/Ki-67 test, and/or Pap cytology result were invited for colposcopy/biopsy to obtain a histopathologic diagnosis (CIN2, CIN3+). Women who showed up for colposcopy but had a normal appearing cervix were not biopsied and were considered as disease negative. Since most women with positive screening test results did not show up for colposcopy as per protocol, a gold standard disease endpoint for the evaluation of the diagnostic performance of the tests was only available for a subset of study participants. Prevalence of positive test results and agreements of the different tests was assessed in the entire study population.
